# Chromosomal mapping of repetitive DNA and retroelement sequences and its implications for the chromosomal evolution process in Ctenoluciidae (Characiformes)

**DOI:** 10.1186/s12862-024-02262-x

**Published:** 2024-05-30

**Authors:** José Francisco de Sousa e Souza, Erika Milena Corrêa Guimarães, Vanessa Susan Pinheiro Figliuolo, Simone Cardoso Soares, Marcelo de Bello Cioffi, Francisco de Menezes Cavalcante Sassi, Eliana Feldberg

**Affiliations:** 1 Conservation and Evolutionary Biology, INPA, National Amazon Research Institute, Av. André Araújo, 2936, Petrópolis, CEP: 69067–375, Caixa Postal 2223, Manaus, 69060-001 Amazonas Brazil; 2 Department of Genetics and Evolution, Federal University of São Carlos, São Carlos, SP Brazil

**Keywords:** Neotropical fish, Pike characins, Sex chromosomes, Chromosomal hotspots

## Abstract

Ctenoluciidae is a Neotropical freshwater fish family composed of two genera, *Ctenolucius* (*C. beani* and *C. hujeta*) and *Boulengerella* (*B. cuvieri*, *B*. *lateristriga*, *B*. *lucius*, *B*. *maculata*, and *B*. *xyrekes*), which present diploid number conservation of 36 chromosomes and a strong association of telomeric sequences with ribosomal DNAs. In the present study, we performed chromosomal mapping of microsatellites and transposable elements (TEs) in *Boulengerella* species and *Ctenolucius hujeta*. We aim to understand how those sequences are distributed in these organisms’ genomes and their influence on the chromosomal evolution of the group. Our results indicate that repetitive sequences may had an active role in the karyotypic diversification of this family, especially in the formation of chromosomal hotspots that are traceable in the diversification processes of Ctenoluciidae karyotypes. We demonstrate that (GATA)n sequences also accumulate in the secondary constriction formed by the 18 S rDNA site, which shows consistent size heteromorphism between males and females in all *Boulengerella* species, suggesting an initial process of sex chromosome differentiation.

## Background

Ctenoluciidae is a Neotropical freshwater fish family composed of two genera: *Ctenolucius* (*C. beani* and *C. hujeta*) and *Boulengerella* (*B. cuvieri*, *B*. *lateristriga*, *B*. *lucius*, *B*. *maculata*, and *B*. *xyrekes*) [1]. Those species have marked characteristics, such as their elongated snout and jaw, the reason why they are popularly known as “bicudas”, “agulhão”, or “pike characins”. They present the dorsal fin located on the back half of the body, which allows representatives of this family to jump out of the water to escape predators, attack small schools of fish, or capture insects that are close to the water [1–3]. .

Cytogenetic investigations in five of the seven valid Ctenoluciidae species revealed a conservation tendency in the diploid number of 36 chromosomes, and a strong association of telomeric sequences with 5 S and 18 S rDNA sites is suggested to be an ancestral characteristic of the family that precedes the divergence of *Ctenolucius* and *Boulengerella* [4, 5]. The accumulation of telomeric sequences associated with the rDNA locus can result in the formation of chromosomal hotspots, which are regions of the genome with a higher frequency of recombination that contribute to generate genetic incompatibilities between populations, which can lead to speciation. Such hotspots also enhance the dissemination of adaptative alleles into a population and can induce the differentiation of sex chromosomes [6–10]. The hotspot region (telomere + 18 S rDNA) in *Boulengerella* species may be identified by traditional Giemsa staining, given the secondary constriction, which also exhibits a size heteromorphism between men and females, indicating a potential XX/XY sex chromosome system [5]. Unlike *Boulengerella* species, which have simple 18 S rDNA sites, the hotspots in the cis-Andean species *C. hujeta* appear to have promoted the dispersion of the 18 S rDNA across the genome, resulting in several chromosomal pairs harboring this motif [4, 5]. However, no cytogenetic data for the trans-Andean species *C. beani* has been reported in the scientific literature, making it difficult to establish if the multiple sites for 18 S rDNA is a plesiomorphic or apomorphic feature of *Ctenolucius.*

The formation and maintenance of chromosomal hotspots can be influenced by several factors, including the association with simple sequence repeats (SSR) and *Rex* retroelements (*Rex*1, *Rex*3, and *Rex*6), which can move and insert in different regions of the genome [11–14]. Microsatellites are SSR that vary from one to six nucleotides, usually presented at telomeric and centromeric regions of autosomal and sex chromosomes in fish genomes, frequently associated with other repetitive DNA sequences [15]. On the other hand, Rex retroelements are non-LTR retrotransposons obtained from the fish model *Xiphophorus* (Teleostei, Poeciliidae) genome, being the Rex3 one of the first examples of a retrotransposon widespread in teleosts [14]. Those retroelements can be presented in specific chromosomal regions/pairs or widely spread among chromosomes, but especially in heterochromatin-rich regions: telomeres, pericentromeres, and centromeres [11, 12]. The mapping of repetitive sequences allowed a broader comprehension of the karyotypic evolution in several fish, such as in *Erythrinus erythrinus* (Characiformes, Erythrinidae), where the colocalization of *Rex*3, 5 S rDNA and (TTAGGG)n in the centromeric position of the large metacentric Y chromosome in karyomorph D was associated with processes that culminate in the differentiation of sex chromosomes [16]. In *Cynodon gibbus* (Characiformes, Cynodontidae), *Rex*6 sequences were associated with the heterochromatinization process of the W chromosome, and probably involved in the dispersion of the microsatellite motifs (CA)15 and (CAC)10, which are accumulated in a small heterochromatic portion of the W, which led to size variations of those sequences by ectopic recombination [17].

Considering that the association of repetitive DNAs in Ctenoluciidae seems to have had a fundamental role in the karyotypic evolution of the family, in the current study we carried out chromosomal mapping of microsatellite and transposable element (TE) sequences in *Boulengerella* species and *Ctenolucius hujeta* to understand how those sequences behave in these organisms’ genomes and their influence on the chromosomal evolution of Ctenoluciidae.

## Results

The chromosomal characteristics, such as the karyotypes and the fundamental numbers (FN) of the species investigated herein, are described in Sousa e Souza et al. (2017; 2021), following 2n = 36 chromosomes, FN = 72 for *Boulengerella* species, and 2n = 36 chromosomes, FN = 68 for *C. hujeta*.

### Retroelements

**Fig. 1:**
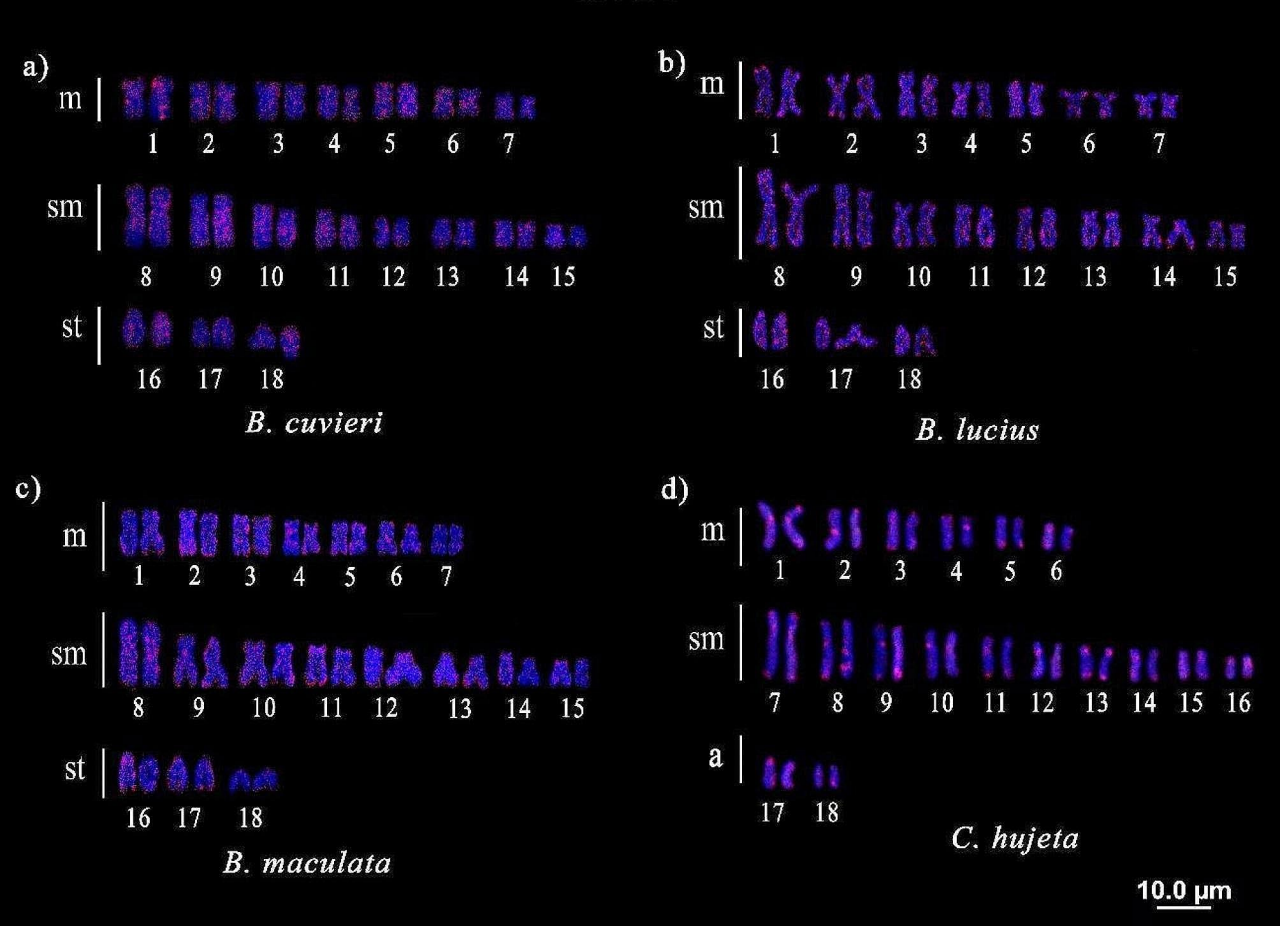
Chromosomal mapping of the TE *Rex*1 in Ctenoluciidae: **a**) *Boulengerella cuvieri*, **b**) *B. lucius*, **c**) *B. maculata*, **d**) *Ctenolucius hujeta*

The TEs *Rex*1, *Rex*3, and *Rex*6 presented a dispersed pattern with a small accumulation in some chromosomes, both in hetero- and euchromatic regions. *Rex*1 (Fig. 1) is present in all species herein analyzed with a dispersed pattern, except for *B. lateristriga*, where no signals were identified (data not shown). On the other hand, *Rex*1 was found in blocks preferentially at the terminal and centromeric regions of most chromosomes in *C. hujeta.*

**Fig. 2:**
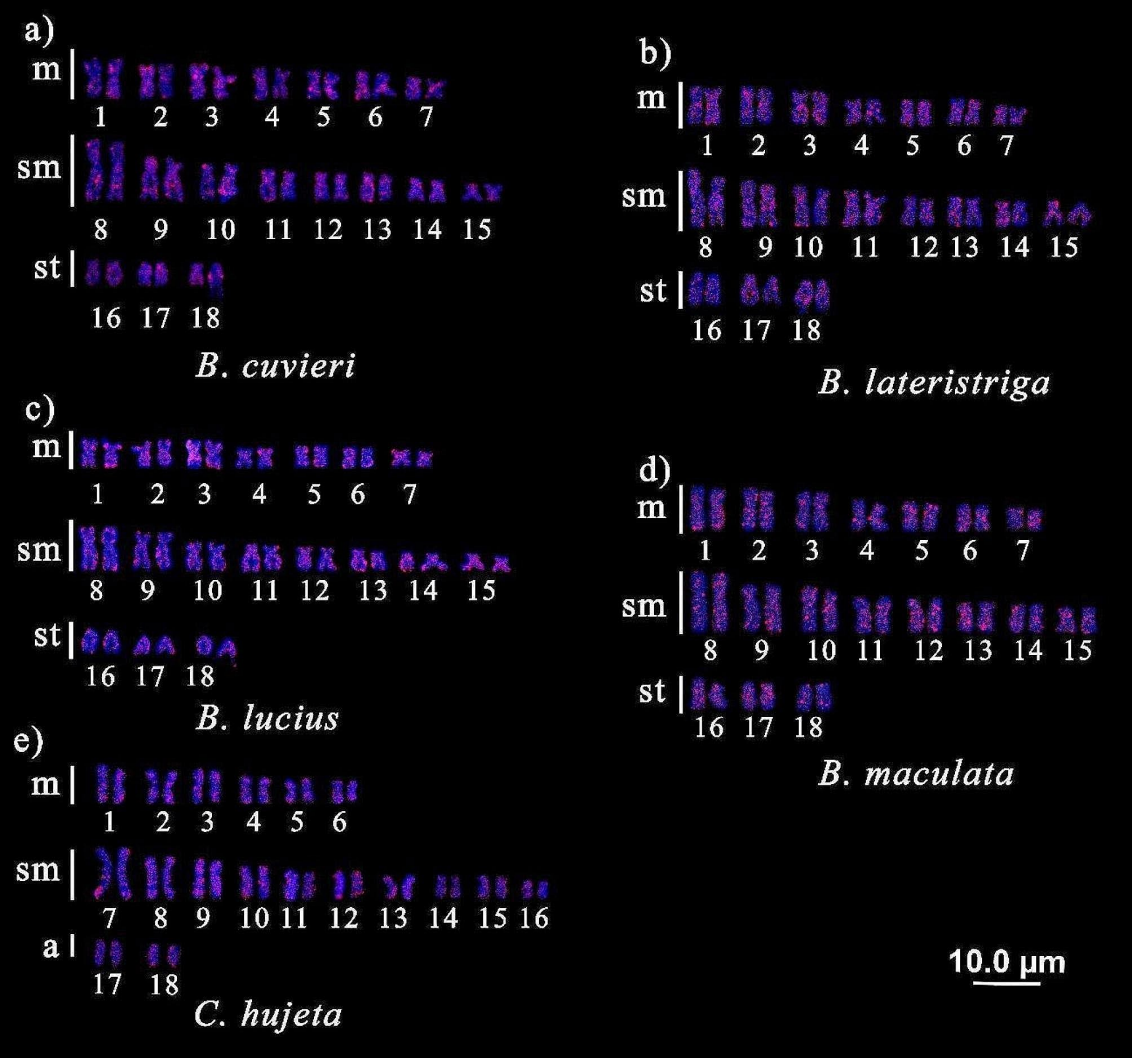
Chromosomal mapping of the TE *Rex*3 in Ctenoluciidae: **a**) *Boulengerella cuvieri*, **b**) *B. lateristriga*, **c**) *B. lucius*, **d**) *B. maculata*, **e**) *Ctenolucius hujeta*

Similarly, *Rex*3 was found dispersed in all chromosomal pairs, forming small blocks in the terminal, pericentromeric, and interstitial regions of some chromosomal pairs in all investigated species, as observed in pair 9 of *B. cuvieri*, pair 15 of *B. lateristriga*, pair 1 of *B. lucius* and *B. maculata*, and pair 12 of *C. hujeta* (Fig. 2).

**Fig. 3:**
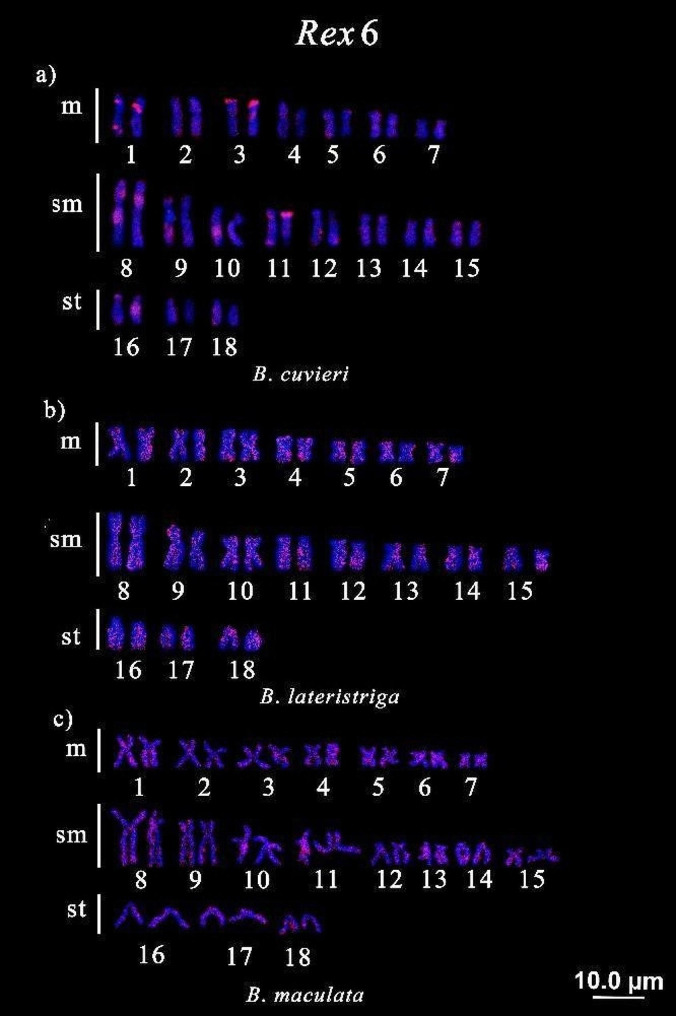
Chromosomal mapping of TE *Rex*6 in Ctenoluciidae: **a**) *Boulengerella cuvieri*, **b**) *B. lateristriga*, **c**) *B. maculate*

*Rex*6 was presented preferentially in conspicuous blocks at the terminal region of pairs 1, 3, and 11 and in the interstitial region at pairs 1, 8, 9, 10, and 16 of *B. cuvieri*. In *B. maculata*, this TE accumulated in the centromeric region of several chromosomes, especially at pairs 1, 3, 6, 8, 10, 11, and 13. On the other hand, in *B. lateristriga*, this TE presented a dispersed pattern in all chromosomes (Fig. 3). Both *B. lucius* and *C. hujeta* do not presented any visible signals (data not shown).

### Microsatellites

**Fig. 4:**
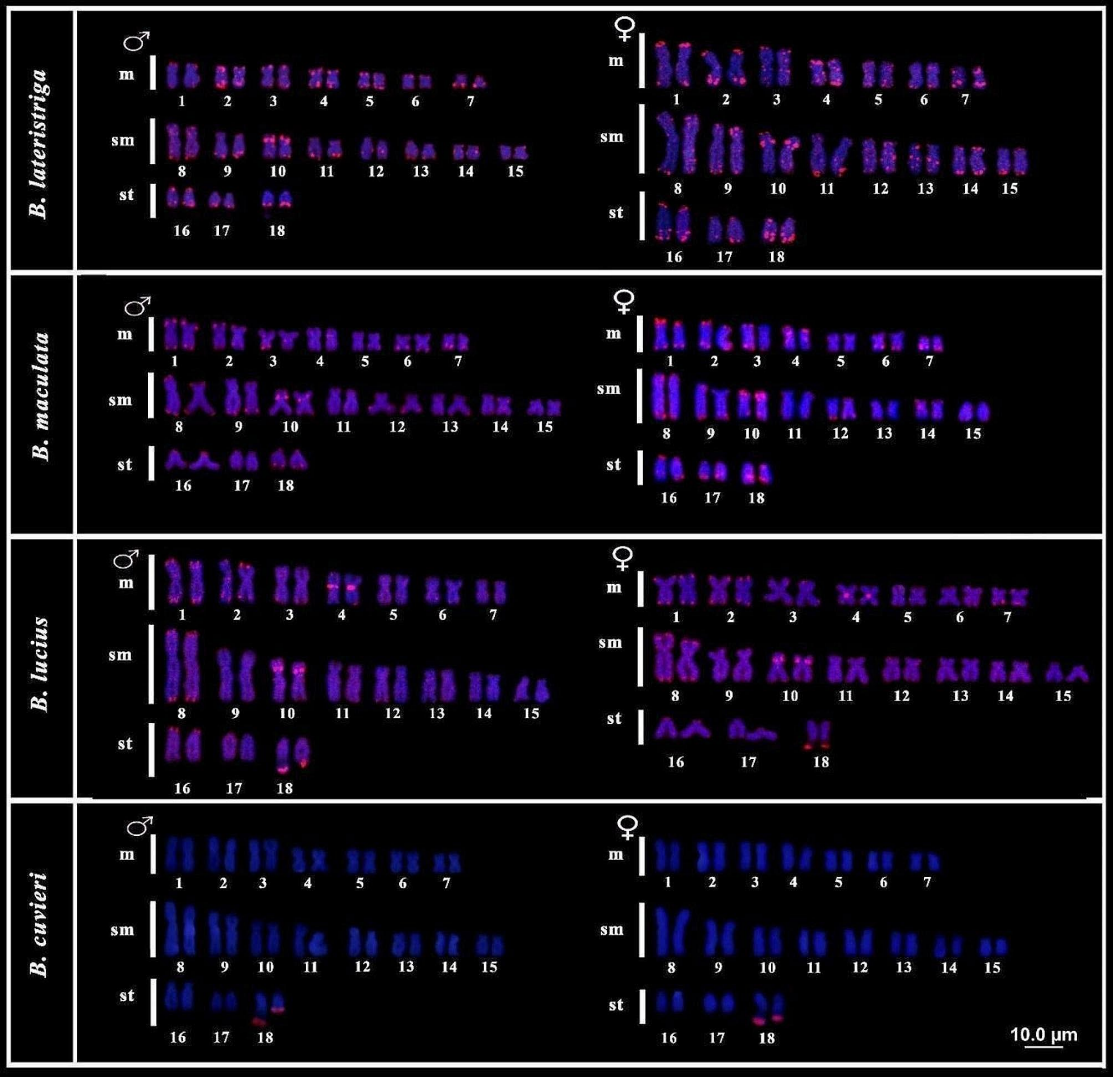
Karyotypes of males and females of *Boulengerella lateristriga*, *B. maculata*, *B. lucius*, and *B. cuvieri*, submitted to FISH with the probe (GATA)_7_. The (GATA)_7_ signals are shown in rhodamine (red) and chromosomes are counterstained with DAPI (blue)

In *B. lateristriga*, *B. maculata*, and *B. lucius*, the microsatellite _d_(GATA)_7_ presented dispersed signals, with preferential accumulation in the telomeric portion of all chromosomes for both males and females, with some chromosomes also presenting interstitial and bitelomeric marks. We highlight the proximal mark in the short arms of pair 10 and the terminal region of long arms at pair 18, which in females seems to be duplicated, and in *B. lucius*, we highlight the species-exclusive centromeric signal on pair 04 (Fig. 4). On the other hand, the microsatellite _d_(GATA)_7_ is present only in pair 18, at the terminal position, in both sexes of *B. cuvieri* (Fig. 4).

**Fig. 5:**
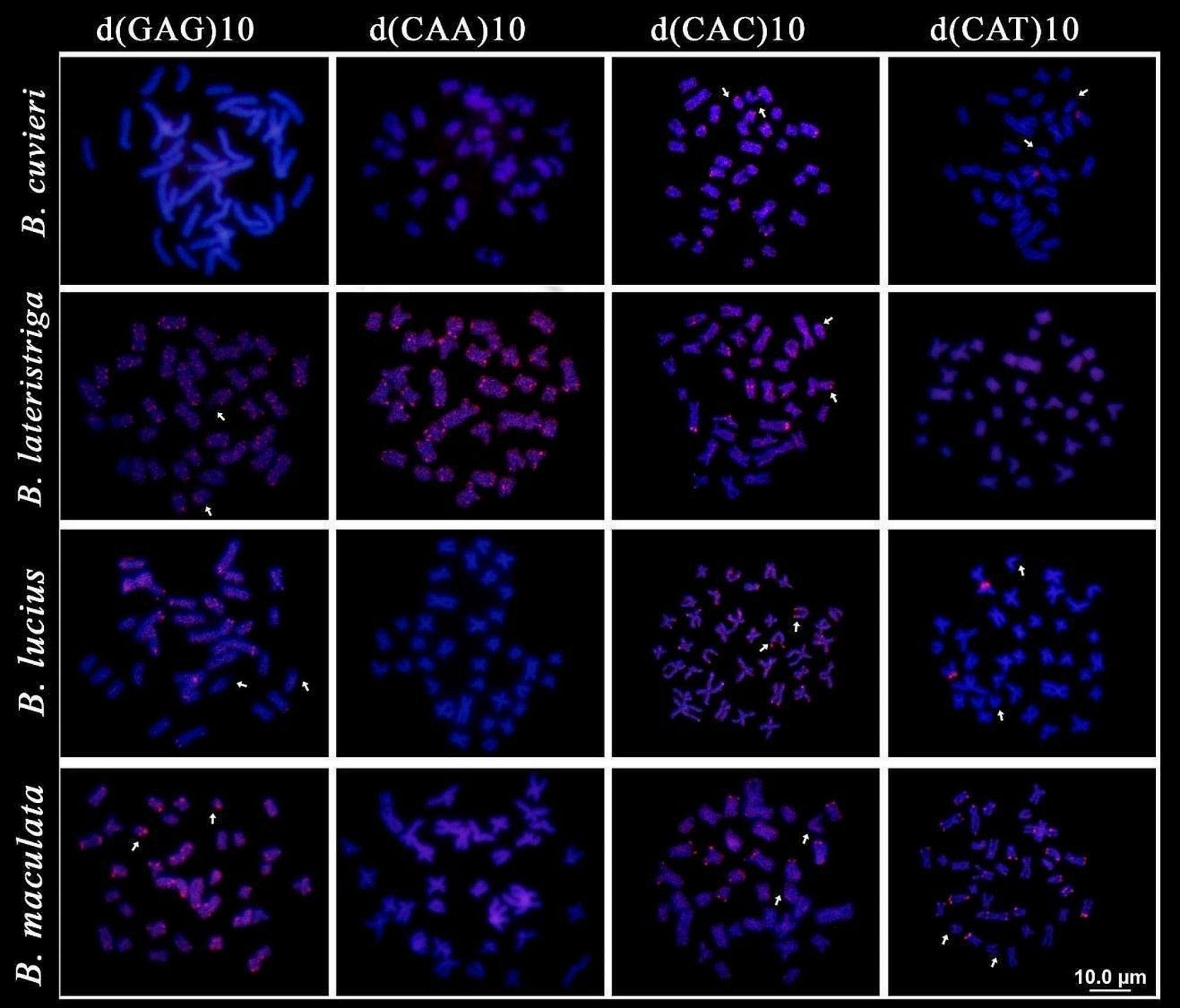
Chromosomal mapping of distinct microsatellite sequences in *Boulengerella cuvieri*, *B*. *lateristriga, B*. *lucius* and *B*. *maculata*. Metaphasic plates hybridized with the microsatellite probes _d_(GAG)_10_; _d_(CAA)_10_; _d_(CAC)_10_ and _d_(CAT)_10_ (red), revealing the distribution pattern on columns 1–4, respectively. Arrows indicate the position of chromosome pair 18

For *B. cuvieri*, no FISH signals were found for the d(GAG)_10_ and d(CAA)_10_ microsatellites. The mapping of the motif _d_(CAC)_10_ revealed telomeric marks in five pairs, and _d_(CAT)_10_ showed telomeric signals in a single chromosome pair (Fig. 5). Moreover, _d_(GAG)_10_ and _d_(CAA)_10_ presented marks with preferential accumulation in terminal and centromeric regions of all chromosomes of *B. lateristriga*. Microsatellite _d_(CAC)_10_ presented terminal marks in two pairs, interstitial on the long arms of the other two pairs, one of which was pair 18 (Fig. 5, arrows). The mapping of the microsatellite d(CAT)10 in *B. lateristriga* did not provide detectable signals. (Fig. 5).

For *B*. *lucius*, the microsatellite _d_(GAG)_10_ presented telomeric marks in four pairs, while the microsatellite _d_(CAA)_10_ was not evidenced. The microsatellite _d_(CAC)_10_ revealed telomeric marks in almost all chromosomes, with accumulation in the secondary constriction of pair 18 (arrow), and _d_(CAT)_10_ showed telomeric marks in a single pair (Fig. 5). Finally, in *B. maculata*, the microsatellite _d_(GAG)_10_ presented bitelomeric marks in most chromosomes, with preferential accumulation at the terminal region of pair 18 (arrow). The microsatellite _d_(CAA)_10_ had no visible signals, while the motif _d_(CAC)_10_ revealed telomeric signals in five pairs, and _d_(CAT)_10_ presented telomeric marks in all chromosomes, in addition to interstitial signals on both long and short arms of a pair, and bitelomeric in three pairs (Fig. 5).

**Fig. 6:**
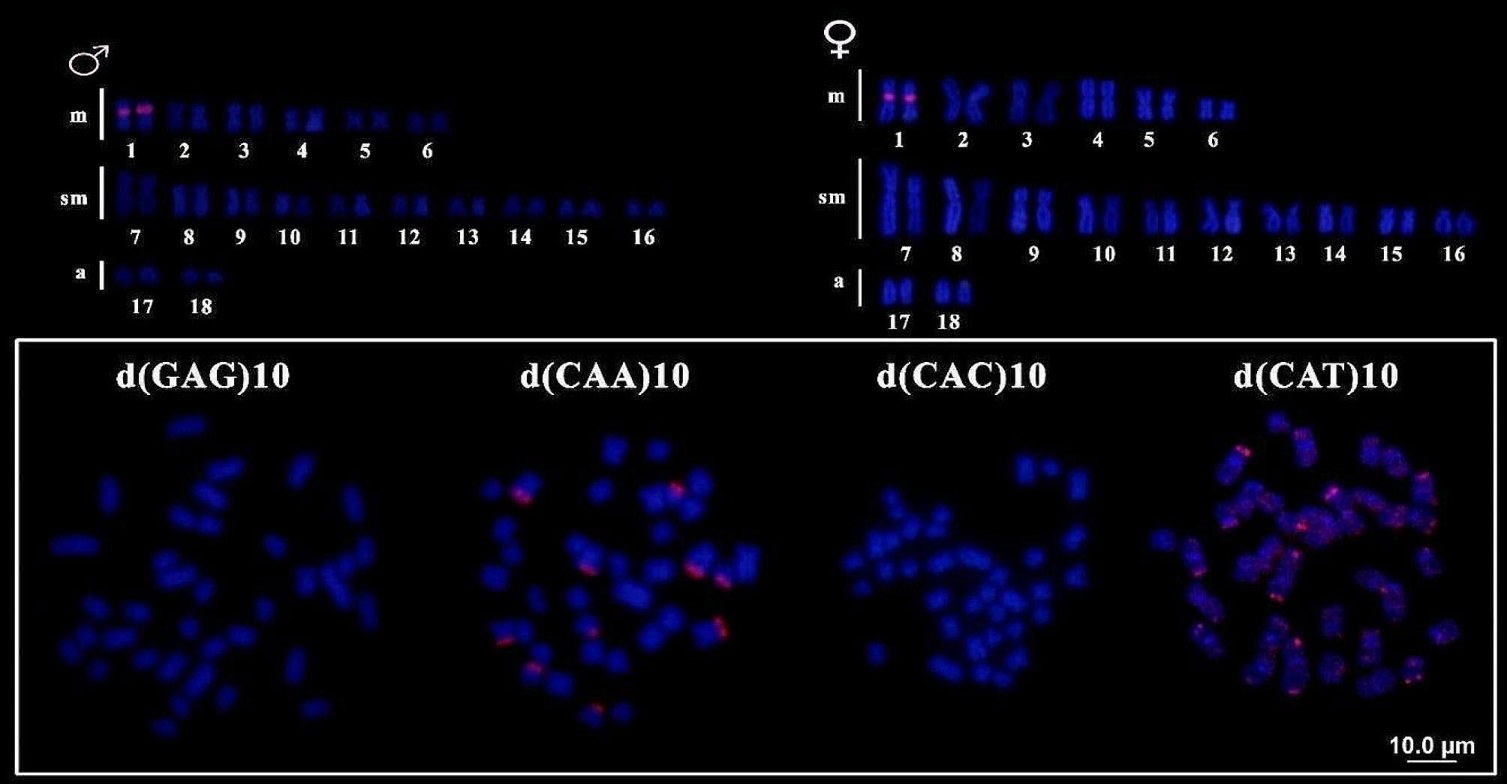
Chromosomal mapping of distinct microsatellite sequences in *Ctenolucius hujeta.* Karyotype of males and females hybridized with microsatellites d(GATA)_7_ (upper section). Metaphasic plates hybridized with microsatellite probes _d_(GAG)_10_; _d_(CAA)_10_; _d_(CAC)_10_; _d_(CAT)_10_, respectively, showing the patter of those SSR at chromosomes (lower section)

For *C. hujeta*, the microsatellite _d_(GATA)_7_ presented marks exclusively in pair 1 at the proximal region of short arms in the chromosomes of both males and females. The microsatellites _d_(GAG)_10_ and _d_(CAC)_10_ were not observed, and the motif _d_(CAA)_10_ showed telomeric marks in five chromosome pairs. The microsatellite _d_(CAT)_10_ presented telomeric marks in most chromosomes (Fig. 6).

## Discussion

According to previous investigations, all Ctenoluciidae species exhibit a similar chromosomal macrostructure, where the conserved status of its diploid number is associated with a notable divergence at the genomic scale, especially regarding repetitive sequences [4, 5]. The current findings allowed us to follow the evolutionary relationships of the family and demonstrate that repetitive DNA sequences have distinct patterns of distribution and accumulation between ctenoluciids. The species-specific distribution of SSRs was observed between *Boulengerella* species and *C. hujeta*, with differences in the number, position, and intensity of hybridization signals for most analyzed motifs. Even in closely related clades or with recent divergence times, differences in the quantity and position of SSRs are common [18–20], as herein observed. Fossil records indicated that ctenoluciids diverged from other lineages at the end of the Miocene (1.8 Ma), probably after the emergence of the Andes Mountains, where large environmental changes occurred [1]. Although recent, such divergence time was enough to be visualized in the chromosomal background of repetitive sequence distribution since species differ in the predominance of several SSRs. For example, d(CAC)_10_ is shared by all *Boulengerella* species but absent in *C. hujeta*, while d(CAA)_10_ only had signals in *B. lateristriga* and *C. hujeta* (Figs. 5 and 6).

The high polymorphism rate of microsatellite sequences, estimated from 10^− 2^ to 10^− 6^*loci* per generation [18, 21, 22], can explain the different distribution patterns between *C. hujeta* and *Boulengerella* species. Microsatellite sequences can expand and contract as a result of ectopic recombination, replication slippage, transposition events or even association with transposable elements (TEs) by a process named “microsatellite seeding” [17, 18, 23, 24]. The dispersed pattern of *Rex*1, *Rex*3, and *Rex*6 and the accumulation at heterochromatic and euchromatic regions of chromosomes syntenic to SSRs suggests that microsatellite seeding is the mechanism responsible for the patterns observed in Ctenoluciidae representants and helps to explain the increase in those sequences in some species. The position of those retroelements in heterochromatic regions is described as an epigenetic mechanism that acts to avoid the excessive propagation of retroelements in the host genome [11, 25, 26], given that the position of those transposable elements in euchromatic regions may generate mutations that affect the levels of genetic expression and the patterns of DNA recombination or even interfere in the organization of genomic architecture [25, 27]. In this sense, we may infer that the position of those TEs in euchromatic regions may have facilitated chromosomal rearrangements such as inversions, deletions, duplications, and translocations, which culminate in karyotypic differences evidenced between *Ctenolucius* and *Boulengerella* [28, 29]. The correlation of environmental conditions and TEs is still a few explored research topic; however, studies have revealed that the distribution of those sequences is susceptible to environmental changes, such as increases or decreases in temperature [12, 30], exposure to pesticides [31] or even in response to stressful and polluted environments [32]. The same also occurs with ribosomal sequences, where differences in the accumulation and distribution of both 5 S and 18 S rDNA were observed inpreserved versus polluted environments [33].

In several fish species, TEs were found associated with ribosomal sequences, as observed in *Hisonotus leucofrenatus* [37], *Erythrinus erythrinus* [38], *Astyanax bockmanni* [35], *Astyanax fasciatus* [39], and several *Ancistrus* species [25]. This kind of transposition occurs given that codificant genes may form complexes with transposable elements and disperse in the species genomes [34-36]. Although not co-hybridized here, it is noteworthy that the secondary constriction formed by the NOR site in *Boulengerella* species (pair 18, according to Sousa e Souza et al., 2017) and *C. hujeta* (pairs 01, 09, and one of the homologs of pair 12, Sousa e Souza et al., 2021) have a small accumulation of Rex1 and Rex3. This same region also harbors (GATA)n sequences, in addition to corresponding to the telomeric region [4, 5]. Besides that, several other microsatellite motifs have been mapped in the secondary constriction, such as d(CAC)_10_, d(CAA)_10_, d(CAT)_10_, and d(GAG)_10_, suggesting that such region might be susceptible to rearrangements and the invasion of repetitive sequences. Considering that pair 18 in *Boulengerella* species is a proto-sex chromosome (Sousa e Souza et al., 2017), such an accumulation reinforces this previous hypothesis. Indeed, the microsatellite (GATA)n is highly conserved between eukaryotes and was isolated from the W chromosome of Caenophidia snakes [43–45]. It is frequently associated with sex chromosomes in mammals and some reptile and fish species [46–51]. In addition to its association with chromatin, transcription factors of heterochromatin, and formation factors, this motif is also present in autosomes, as already reported in some fish species [19, 20], presenting regulatory functions in the process of sex chromosome differentiation [19, 49]. The (GATA)n microsatellite is located in the same chromosomal pair that also carries the 5 S rDNA in *Boulengerella* species, with the accumulation of this SSR in the pericentromeric region of pair 10 in *B. lateristriga*, *B. lucius*, and *B. maculata* and in pair 4 of *B. lucius*. Considering that the first chromosome pair of all *Boulengerella* species and *Lebiasina bimaculata*, *L. melanoguttata*, and *L. minuta* are homeologs [52, 53] and carry at least one copy of the 5 S rDNA, this may correspond to the plesiomorphic carrier of the 5 S rDNA. Given that this region presents an accumulation of SSR and Rex retroelements, we can consider this region also a chromosomal hotspot. It is known that copy and paste events mediated by Rex retroelements may carry other ribosomal sequences, as already reported for *Erythrinus erythrinus* [16, 54], *Pyrrhulina australis, Pyrrhulina* aff. *australis* [55], and *P. brevis* [56].

The characterization of chromosomal hotspots might help the elucidation of the evolutionary pathways that led to differences in the karyotypes of *Boulengerella* species and *C. hujeta*. Although the synteny of telomeric, SSRs, *Rex*, and ribosomal sequences cannot be established by the current data, such an association might be further investigated. Notably, the pair that carry such sequences in *Boulengerella* species presents a size heteromorphism between males and females. It is worth noting that size heteromorphism in the secondary constriction, or of the nucleolar pair, is common in fish, occurring in both sexes and, for this reason, not associated with sex chromosomes [40–42]. However, in *B. cuvieri, B. lateristriga, B. lucius*, and *B. maculata*, this heteromorphism occurs only in the secondary constriction of males, a fact that led us to hypothesize that the accumulation might be a result of the arose of sex-specific sequences expressed in males with the absence of the secondary constriction in one of the pair 18 homologs. In this context, besides the investigation of synteny between the repetitive sequences abovementioned, more refined analysis such as comparative genomic hybridization (CGH) or even whole chromosome painting (WCP), may be useful tools to confirm the presence or absence of sex-specific sequences associated with pair 18 in *Boulengerella* species.

The conservation of diploid numbers with variation in the distribution of repetitive sequences is not an exclusive feature of Ctenoluciidae members. Other freshwater fish groups, such as the diploid lineages of Cyprinidae, share a common 2n = 50 but with divergence in repetitive DNA distribution (e.g [57]). . . Similarly, Carangidae marine fishes present a conserved 2n = 48, with variation in the proportion of bi-armed and acrocentric chromosomes [58]. For marine fishes, the maintenance of gene flow between large areas of species distribution is accepted as the main explanation for why the 2n is conserved [59]. However, such an explanation is evoked for groups where the divergence of the clades is old, which is not the case of ctenoluciids. In this scenario, it is possible that the recent divergence time was insufficient to generate large-scale changes in karyotype macrostructure, or at least those that can be visualized by classical cytogenetics. Indeed, molecular cytogenetics procedures allowed better access to the genome mainly in groups with conservative karyotypes [60], as in Ctenoluciidade, although small differences could already be observed in C-banding [5]. There is not a complete explanation for why some groups retain a conserved diploid number and others exhibit a wide variation. Some authors suggest that the plasticity of fish genomes allows structural variations without hampering their proper function or segregation [61]. While this question remains a mystery, increasing the knowledge on repetitive DNA distribution and possible evolutionary targets for karyotype organization allows a comprehensive view of Ctenoluciidae evolution.

## Materials and methods

Species, sample points, and number of investigated specimens are described in Table 1. The samples herein analyzed correspond to the same ones previously described in Sousa e Souza et al. (2017).

**Table 1:** Species, sex, number of specimens, sample points, and Voucher of the investigated individuals

Species	Specimens	Locality	Voucher
*Boulengerella cuvieri*	6 ♂ and 8 ♀	Rio Negro, Amazonas, Manaus, Brasil (3°10′30.8′′S, 59°56′30.3′′W)	INPA-ICT 053247
*Boulengerella lateristriga*	7 ♂ and 7 ♀	Novo Airão, Amazonas, Manaus, Brasil (2°37′28.5′′S, 60°58′16.8′′W)	INPA-ICT 053246
*Boulengerella lucius*	6 ♂and 6 ♀	UHE Balbina, Amazonas, Brasil (1°55′02.2′′S, 59°28′23.7′′W)	INPA-ICT 053248
*Boulengerella maculata*	7 ♂ and 8 ♀	Anavilhanas, Amazonas, Brasil (2°33′28.4′′S, 60°46′29.7′′W)	INPA-ICT 053249
*Ctenolucius hujeta*	6 ♂ and 4 ♀	Aquarist RsDiscus aquarium and ornamental fish	INPA- ICT 059514

In summary, fish were kept in aerated aquariums and, after 12 h of biological yeast stimulation [62], were euthanized in a 10% Eugenol solution, following the recommendations of the CONCEA Euthanasia Practice Guideline [63]. Then, mitotic metaphases were obtained from anterior kidney cells after in vitro treatment with colchicine [64, 65].

### Probe preparation and FISH

The genomic DNA, used for all methodological steps, was extracted from muscle tissues fixed in 96% ethanol, according to the protocol established by the extraction kit Wizard® Genomic DNA Purification (Promega Corporation, Wisconsin, USA). The extracted DNA was subjected to electrophoresis in a 1% agarose gel and stained with Gel Red (Biotium) to check integrity. The following microsatellite sequences were used as probes: d(GATA)_7_, d(CAC)_10_, d(CAA)_10_, d(CAT)_10_ and d(GAG)_10_, since they correspond to standard microsatellite sequences used in fish cytogenomics studies [15]. These sequences were directly labeled with Cy3 in the 5’ end during synthesis by VBC-Biotech (Viena, Austria), following [66]. The retroelements *Rex*1, *Rex*3, and *Rex*6 were amplified by polymerase chain reaction (PCR) from the *Boulengerella lateristriga* genome using the following primers: RTX1-F1 (5’-TTC TCCAGTGCCTTCAACACC) and RTX1-R1 (5’-TCCCTCAGCAGAAAGAGTCTGCTC) [67]; RTX3-F3 (5’- AACACCTTGGCTGCGCCTAG) e RTX3-R3 (5’-TTGAGG AACCGACGCGGATC) [68]; *Rex*6-Medf1 (5’-TAAAGCATACAT GGAGCGCCAC) and *Rex*6-Medf2 (5’-AGGAACATGTGTGCA GAATATG) [15].

Fluorescence in situ hybridization (FISH) was based on the protocol of [69], with some modifications. Slides were washed in PBS 1X solution for 5 min and fixed in 1% formaldehyde for 10 min. Next, the slides were washed again in PBS 1X solution for 5 min and dehydrated in an ethanol series (70, 85, and 100%) for 5 min at each concentration. After drying, the slides were denatured in formamide 70%/2xSSC at 70 °C and dehydrated in an ethanol series (70, 85, and 100%) for 5 min each. Next, the denaturation of the hybridization mix (1 µL of the labeled probe + 20 µL of DS solution per slide) occurred in a thermoblock for 10 min at 99 °C. After denaturation, the probe was applied to each slide and incubated in a moist chamber at 37 °C for 24 h to allow hybridization. Chromosomes were counterstained with DAPI (1,2 µg/mL), and the slides were mounted in an antifade solution (Vector, Burlingame, CA, USA).

### Chromosomal analysis

At least 20 metaphases of each specimen were investigated to confirm the chromosomal distribution of retroelements *Rex*1, *Rex*3, and *Rex*6 and microsatellite sequences. Images were obtained using an Olympus BX51 (Olympus Corporation, Ishikawa, Japan) equipped with CoolSNAP. The best metaphases were captured, and karyotypes were assembled in Adobe Photoshop CS6 software. Chromosomes were classified as metacentric (m) or submetacentric (sm), following the classification proposed by [70], given the arm ratio.

## Conclusion

Notably, the conservatism in Ctenoluciidae karyotypes is restricted only to the diploid number, and in this sense, we may infer that the diversity of SSR and *Rex*1, *Rex*3, and *Rex*6 retroelements evidenced in these species was essential to the formation of hotspot chromosomal regions. Such a region may have led to chromosomal rearrangements, as observed by the presence of acrocentric chromosomes, multiple sites of 18 S rDNA, chromosomal heteromorphisms, or even the transposition of 5 S rDNA sequences in the same genus, as observed in *B. lucius* when compared to other *Boulengerella* species.

The chromosomal hotspot formed by the association of several repetitive DNAs (e.g., 5 S/18S rDNA, (TTAGGG)n, d(GATA)_7_, d(CAC)_10_, d(CAA)_10_, d(CAT)_10_, d(GAG)_10_, and *Rex* sequences) in *C*. *hujeta* led to a dispersion of 18 S rDNA sites to pair 01 and to one of the homologs of pair 12, while in *Boulengerella* species, the same chromosomal hotspot at pair 18 induced the rise of a size heteromorphism in the secondary constriction between male and female that was associated with the syntenic accumulation of (GATA)n sequences, which may indicate an initial process of sex chromosome differentiation, which needs to be further investigated.

## Data Availability

The datasets generated and/or analyzed during the current study were deposited in the National Amazon Research Institute (INPA) Fish Collection under the following numbers: *Boulengerella* (INPA-ICT 053246, 053247, 053248, 053249, and 053250) *Ctenolucius hujeta* (INPA-ICT 059514). https://portalcolecoesdb.inpa.gov.br/ictiologia/.
